# Sub-23 nm Particles Dominate Non-Volatile Particle Number Emissions of Road Traffic

**DOI:** 10.1021/acs.est.3c03221

**Published:** 2023-07-14

**Authors:** Henna Lintusaari, Heino Kuuluvainen, Joonas Vanhanen, Laura Salo, Harri Portin, Anssi Järvinen, Paxton Juuti, Riina Hietikko, Kimmo Teinilä, Hilkka Timonen, Jarkko V. Niemi, Topi Rönkkö

**Affiliations:** † Aerosol Physics Laboratory, Physics Unit, Tampere University, Tampere 33720, Finland; ‡ Airmodus Oy, Helsinki 00560, Finland; § Helsinki Region Environmental Services Authority, Helsinki 00240, Finland; ∥ Atmospheric Composition Research, Finnish Meteorological Institute, Helsinki 00560, Finland

**Keywords:** ultrafine particle, nanoparticle, urban pollution, air quality, street canyon, emission factor

## Abstract

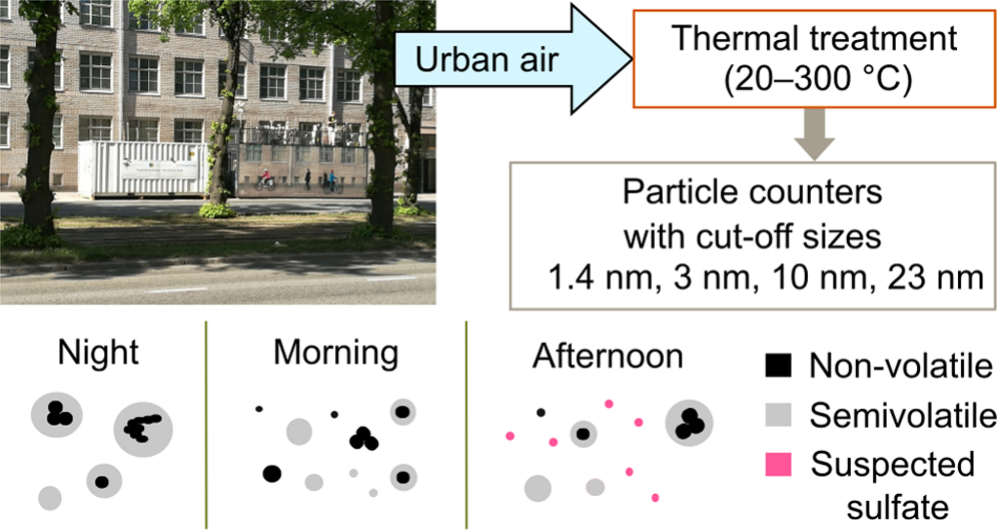

Ultrafine particles (<100 nm) in urban air are a serious health hazard not yet fully understood. Therefore, particle number concentration monitoring was recently included in the WHO air quality guidelines. At present, e.g., the EU regulates particle number only regarding the emissions of solid particles larger than 23 nm emitted by vehicles. The aim of this study was to examine the non-volatile fraction of sub-23 nm particles in a traffic-influenced urban environment. We measured the number concentration of particles larger than 1.4, 3, 10, and 23 nm in May 2018. Volatile compounds were thermally removed in the sampling line and the line losses were carefully determined. According to our results, the sub-23 nm particles dominated the non-volatile number concentrations. Additionally, based on the determined particle number emission factors, the traffic emissions of non-volatile sub-10 nm particles can be even 3 times higher than those of particles larger than 10 nm. Yet, only a fraction of urban sub-10 nm particles consisted of non-volatiles. Thus, while the results highlight the role of ultrafine particles in the traffic-influenced urban air, a careful consideration is needed in terms of future particle number standards to cover the varying factors affecting measured concentrations.

## Abstract 2

The role of sub-23 nm particles in urban air quality is easily underestimated. This study reports that the sub-23 nm particles dominate the non-volatile particle number emissions of road traffic in an urban traffic-influenced environment.

## 1 Introduction

Urban cities are typical settings where high air pollution levels meet high population density. As one significant air quality factor, fine particulate matter (PM_2.5_) has been estimated to cause 3.3–10.2 million premature deaths worldwide annually.^1−3^ Along with mortality, particulate pollution is linked to various adverse health effects such as respiratory and cardiovascular diseases.^4,5^ Health impacts depend on particle characteristics including size, shape, and chemical composition.^6^ In contrast to larger-sized particles, ultrafine particles (UFPs, <100 nm) efficiently deposit in all regions of the respiratory track and get access to the blood circulation resulting in distribution throughout the body.^7^ Small particles may enter the brain directly via the olfactory bulb.^8^ For example, in a study by Weichenthal et al.^9^ UFPs were associated with brain tumor incidences, whereas no association was found between PM_2.5_ and increased incidence of brain tumors. Moreover, there is an indication that particles smaller than 20 nm can end up into fetuses from the blood circulation of pregnant rats.^10^ At the same time, UFPs have a greater surface area per mass unit, meaning a high capacity to carry toxic compounds into the human body.^11^

Instead of mass-based examination, the particle surface area and number appear to better predict health effects of UFPs.^7^ Whereas UFPs dominate the number concentrations,^12^ the mass of UFPs is negligible when compared to larger particles meaning that UFPs are basically ignored when only mass-based metrics are used. In 2021, World Health Organization (WHO) introduced a good practice statement recommending measurement of the particle number concentration as a part of ambient air quality monitoring.^13^ There, a lower size limit for measured particles is set to ≤10 nm and indicative values for low and high particle number concentrations are <1000 #/cm^3^ and >10,000 #/cm^3^ (24 h mean), respectively. Obligation to measure the particle number concentration with a lower limit of ≤10 nm is also included in the latest European parliament proposal for a directive on air quality issued in 2022.^14^

In contrast, the European Union introduced a particle number emission limit for solid particles emitted by vehicles as a part of European emission standards already in the past decade. The limit was first implemented for diesel passenger cars (Euro 5b) in 2011, following a limit for heavy-duty engines (Euro VI) in 2013 and gasoline passenger cars (Euro 6) in 2014. However, particle number measurements follow the Particle Measurement Program (PMP) protocol that considers only solid particles above 23 nm in size.^15^ Even though sub-23 nm emission particles are in many cases known to be volatile, non-volatile particles have also been observed in this size range.^16−18^ This has led to further study, technical development, and discussion on the feasibility to regulate particle number emissions in the sub-23 nm region down to at least 10 nm.^19^ Following, the EU has recently proposed a lower limit of 10 nm as a new basis for particle number emission regulation in Euro 7 standard entering into force in 2025 (Euro 7).^20^ All the same, it has been found that the size range of traffic emitted particles extends even down to 1–3 nm.^21^

The aim of this study was to examine the non-volatile fraction of sub-23 nm particles in a traffic-influenced urban environment. We used a condensation particle counter battery^22^ (CPCB) to measure simultaneously the number concentration of particles larger than 1.4, 3, 10, and 23 nm with 1 s time resolution for one month in May 2018. Volatile compounds were removed from particles in the sampling line using a combination of a hot and a cold ejector diluter. Finally, we determined the typical number concentration of non-volatile particles, size ranges in which non-volatile particles are found, their emission factors (EFs), and the proportion of non-volatile particles to the total particle number in traffic-influenced urban air.

## 2 Methods

### 2.1 Measurement Site and Conditions

The measurements were carried out in a street canyon (Mäkelänkatu 50; 60.19654N, 24.95172E) on a busy street leading toward the city center of Helsinki, Finland, in May 2018. Measurement devices were installed in a sea container, owned by Finnish Meteorological Institute, that was situated next to an urban supersite air quality measurement station (denoted later as Supersite) operated by Helsinki Region Environmental Services Authority (HSY). The sea container and the Supersite were located on the pavement within less than 0.5 meters of lanes. The traffic rate of the street is ∼28,000 vehicles/weekday of which the heavy-duty vehicles account for 10% (City of Helsinki). In 2018, Finland’s motor vehicle stock comprised approximately 71% gasoline and 28% diesel passenger cars, while 98% of heavy-duty vehicles were diesel vehicles.^23^

The street consists of two pavements, six lanes, and two tramlines lined with trees in the middle, giving a total width of 42 m. The height of surrounding buildings is 16–19 m leading to an average height-to-width ratio (H/W) of 0.45. Perpendicular wind directions allow the formation of a street canyon vortex,^24^ but the low H/W ratio is not ideal for vortex formation. Especially in low wind speed conditions, the vortex may play a minor role in the dispersion compared to the turbulent mixing caused by traffic.^25^ The pollution levels are highest at the street level addressing well the direct effects of traffic. The site and its air flow patterns are described in more detail by Järvi et al.,^24^ Kuuluvainen et al.,^25^ and Olin et al.^26^

The ambient temperature during the measurements varied from 3.2 to 28.1 °C with a relatively cold and humid first 2 weeks followed by a warm and dry rest of the month. Daily mean, minimum, and maximum values for temperature and relative humidity are presented in Figure S4.

### 2.2 Volatility Condensation Particle Counter Battery

**Figure 1:**
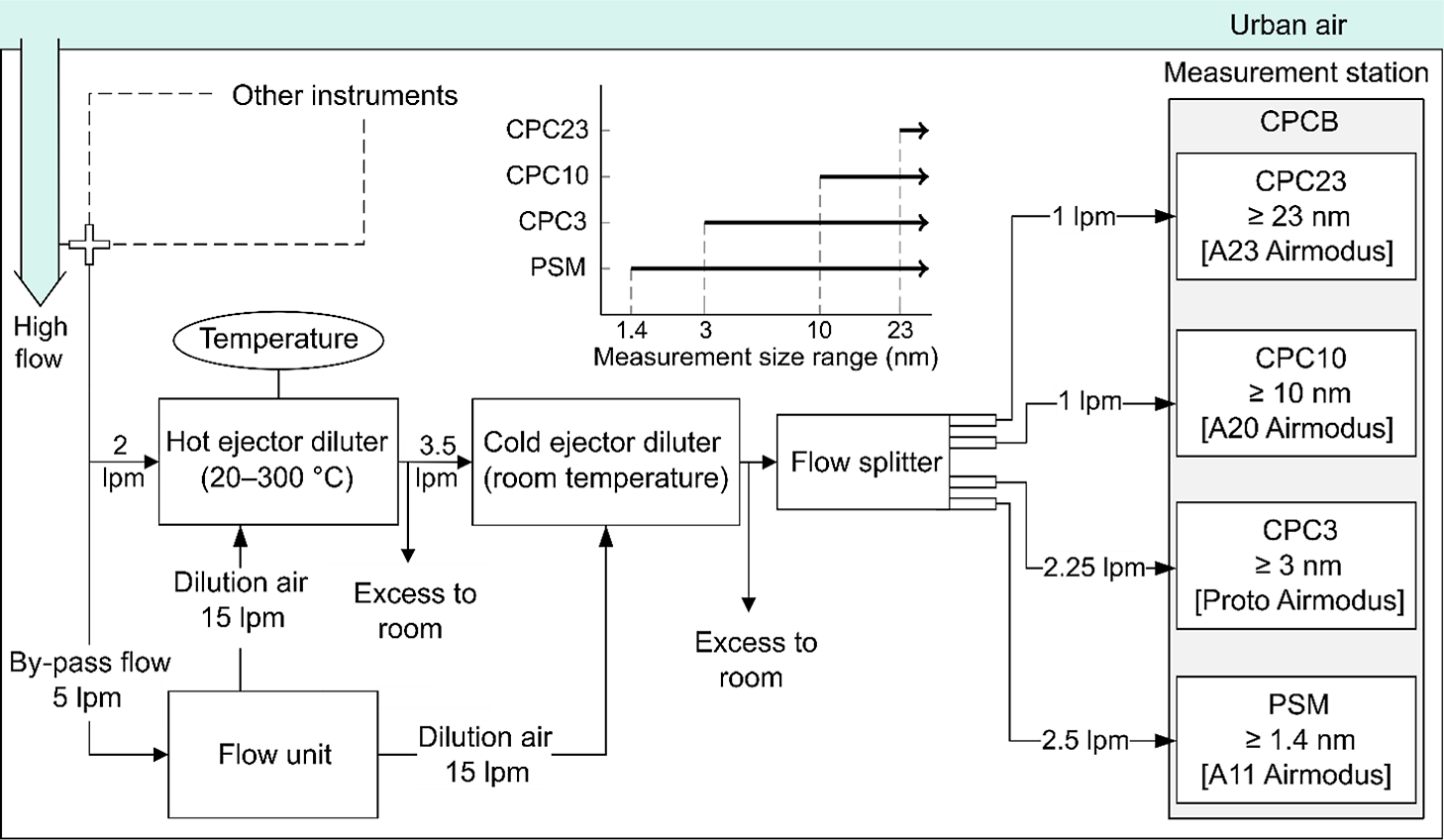
Volatility CPCB measurement setup. The sample air was drawn from the roof into the measurement station, diluted with two ejectors, and split to multiple CPCs and a PSM. First ejector was heated to desired temperature between 20 and 300 °C in order to remove a certain volatile fraction of the ambient air. Particle number size ranges measured by CPCB are illustrated by graph in the middle.

The measurement setup consisted of a CPCB: three condensation particle counters (Airmodus A23 CPC, Airmodus A20 CPC, modified Airmodus A20 CPC^27^) with different cutoff sizes and a combination of a particle size magnifier and a condensation particle counter (Airmodus A11 nCNC^28^) in parallel. The cutoff sizes (defined here as the minimum particle diameter of detection at which the counting efficiency is 50%) of the instruments were 23, 10, 3, and 1.4 nm, respectively. Hence, the instruments are here referred to as a CPC23, a CPC10, a CPC3, and a PSM. The instruments measured the total particle number concentration simultaneously with a time resolution of 1 s, thus the number concentration of size ranges of 1.4–3, 3–10, and 10–23 nm could be obtained with the same time resolution from the difference of two instruments as illustrated in Figure 1.

The aerosol sample was taken from the roof of the measurement station, 4 m above the ground level, through a probe with an air blower causing a high suction flow (690 liters per minute). The sample was then led through a branch above the blower, split twice—first to other instruments and then to bypass—and finally led further to the CPCB through a PMP^15,29^-like sampling system. The sampling line included a hot ejector diluter in which the sample was heated to desired temperature between 20 and 300 °C. The sample was considered non-volatile when the sample temperature in the hot ejector was approximately 300 °C (290–330 °C) and ambient for temperatures less than 35 °C. The temperature limit of 300 °C for the definition of non-volatile particles was adopted from the EU emission legislation following the PMP^15,29^ protocol. Hot diluter was followed by another ejector diluter operating at room temperature. Thermal treatment with a combination of two ejectors was chosen to keep line losses at minimum as sub-23 nm particles get easily lost in sampling. After the dilution, the sample was split to CPCB instruments with a flow splitter. The setup is illustrated in Figure 1 and is henceforth referred to as a volatility CPCB.

The hot ejector was mainly used with a fixed temperature of 300 °C following the measurement matrix presented in Table S1. During the last measurement week (22–28 May), the hot ejector heater was programmed to automatically turn on for 6 min and then turn off for 15 min. This on–off loop created a cycle comprising approximately 3 min of measurements at the temperature minimum (room temperature), 3 min at the maximum (300 °C), and 15 min during the temperature transition. To gain more data from intermediate temperatures, additional step measurements were conducted on May 22 and 25. The temperature was then adjusted manually to 300, 265, 150, 80, and 50 °C and concentrations were measured with each temperature for 3 min repeating four times.

Diffusional particle losses in the sampling probe were estimated to be minimal due to the high flow rate. A bypass flow of 5 liters per minute was added before the hot ejector to reduce sample losses between the probe and the hot ejector. Losses of the sampling line were measured in a laboratory producing 5–50 nm silver particles with a tube furnace after the campaign (see the Supporting Information). Penetration efficiencies for different particle size ranges at different ejector temperatures were determined from theoretical fits of the measurement results. When measuring sub-23 nm particles, the minimization and determination of sampling losses play a crucial role. Hence, the full loss correction procedure is described in detail in the Supporting Information and the volatility CPCB results presented later are loss-corrected accordingly. Also, the dilution ratio (DR = 33–38) of the sampling line was considered (see the Supporting Information).

### 2.3 Sub-3 nm Measurements of the Volatility CPCB

When the sample was heated to 300 °C, we observed an artifact in sub-3 nm size range, but it was reduced by lowering the PSM saturator flow from 1.3 liters per minute to 1 liter per minute. With the highest saturator flow rate setting of 1.3 liters per minute, the PSM might already detect large molecules that are evaporated but not removed by the hot ejector diluter. Hence, the reliable weekday measurement data for sub-3 nm size range were limited to a 3 day period from May 24 to 28, 2018 with a maximum artifact of a few hundred particles per cubic centimeter.

The PSM was calibrated with NiCr oxide particles which behave similarly as ash particles and thus correspond reasonably with the non-volatile particles measured in the traffic-influenced environment of this study. Nevertheless, there is an uncertainty in determining the cutoff size as, e.g., a shift of +0.3 nm in diameter has been previously suggested when measuring organic compounds.^30^ The sub-3 nm size range is also challenging with respect to the particle line losses (see the Supporting Information). More discussion on the sources of uncertainty in the sub-3 nm particle concentration measurement is found in Kangasluoma and Kontkanen.^31^

### 2.4 Additional Measurements

The ambient particle number concentration (meaning here all particles regardless of their volatility) was simultaneously measured by another set of CPCB (CPCB-2) with cutoff diameters 1.2, 3, and 7 nm (Airmodus A11 nCNC, TSI Ultrafine CPC 3776, and Airmodus A20 CPC). CPCB-2 was installed in the same measurement station and drew sample air from a similar probe as the volatility CPCB. Lines after the probe were as short as possible. Line losses of CPCB-2 are not considered in the results. The ambient particle number concentration was also measured by a differential mobility particle sizer^32^^,p. 349^ (DMPS) located in the Supersite. The DMPS comprised a Vienna-type differential mobility analyzer and an Airmodus A20 CPC. The DMPS measured particle number size distribution with 9 min scans covering the size range of 6–800 nm. In the results, the DMPS measurements are corrected for line losses using theoretical estimation. In addition to particles, CO_2_ (LI-COR LI-7000) and NO_*x*_ (Horiba APNA 370) concentrations were measured at the Supersite.

### 2.5 Emission Factor Calculation

EFs are relations between pollutant emissions and the activity causing them. Road vehicle EFs are typically derived for vehicle categories but they can also be implemented as averages for an entire fleet.^33^ In this study, the particle number EFs represent the influence of the entire fleet of vehicles passing the measurement station where the air corresponds to a sample in between tailpipe aerosol and diluted background aerosol. Therefore, particles are assumed to disperse similarly as CO_2,_ and an EF of CO_2_ determined for average Finnish road traffic^34^ is applied in the calculation of EFs.

**Figure 2:**
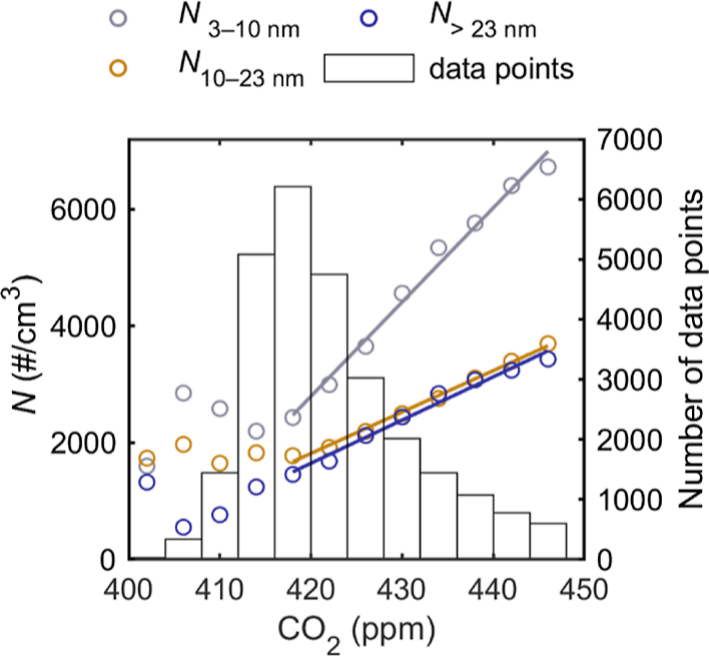
Particle number concentration (*N*) of non-volatile particles in different particle size ranges as a function of the simultaneously measured CO_2_ concentration. Circular markers represent averages of number concentrations in 4 ppm CO_2_ intervals. Right *y* axis represents the number of data points in each CO_2_ interval in the histogram. Linear fits are applied to markers having at least 100 data points and CO_2_ values above the background level.

In the approach similar to previous studies,^21,35^ particle number concentrations are first combined with simultaneously measured CO_2_ data. Then, the particle number concentrations are averaged over 4 ppm CO_2_ intervals and a linear fit is applied to calculated averages. In this study, the measured particle number concentrations were log-normally distributed and thus the geometric mean was used for averaging (see the Supporting Information). Figure 2 presents an example of averaged non-volatile particle number concentrations of different size ranges as a function of the CO_2_ concentration. With low CO_2_ concentrations, there are some variations in the number concentration. As the CO_2_ increases above a background limit, an evident linear correlation is observed and fits for each size range are applied. In this study, limit for background CO_2_ was determined from the 20th percentile of CO_2_. The amount of data points in each CO_2_ interval is also presented in Figure 2. Averages below the background CO_2_ level or having less than 100 data points were excluded when applying linear fits.

The EF of the particle number concentration (EF_N_) is then calculated using the EF of CO_2_ and the slope of the corresponding fit. Assuming ideal gas law and standard conditions (STP), the relation follows as
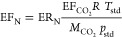
1where ER_N_ is the slope of the particle number concentration as a function of the simultaneously measured (as ppm) CO_2_ concentration (also known as the emission ratio), EF_CO_2__ is the EF of CO_2_ [3141 g CO_2_/(kg fuel)],^34^*R* is the molar gas constant [8.3144621 J/(K mol)], *T*_std_ is the standard temperature (273.15 K), *M*_CO_2__ is the molar mass of CO_2_, and *p*_std_ is the standard pressure (10^5^ Pa). The average temperature during different measurement periods varied from 14.2 to 17.6 °C. As the temperature differed from standard conditions, the number concentrations were normalized to standard conditions before determining the EF.

## 3 Results and Discussion

### 3.1 Ambient Distribution

**Figure 3:**
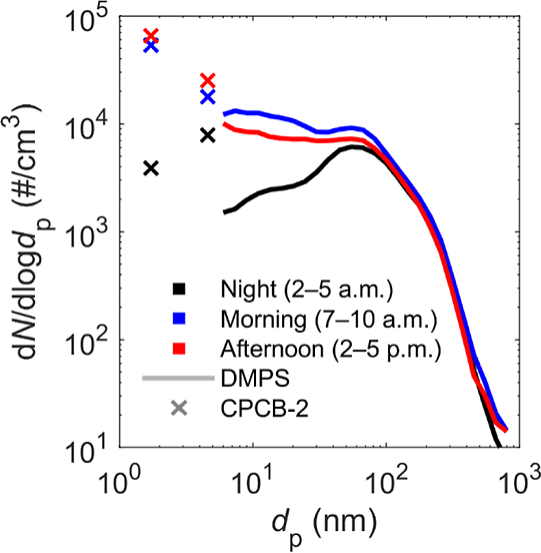
Ambient particle number size distribution in the street canyon on weekdays. Three different times of the day, night (black), morning (blue), and afternoon (red) are distinguished from the data set. Data was collected on weekdays during Apr 27 to May 31, 2018 of which the DMPS measured the whole period with 9 min scans and CPCB-2 instruments measured with 1 s resolution with following coverages: *N*_>7nm_ 80.3%, *N*_>3nm_ 61.3%, and *N*_>1.2nm_ 40.15%.

The average ambient particle number size distribution measured by the DMPS and CPCB-2 in the street canyon on weekdays is shown in Figure 3. In the figure, different hours of the day are segregated to represent night (2 to 5 a.m.), morning (7 to 10 a.m.), and afternoon (2 to 5 p.m.). Each number size distribution is derived by calculating a diurnal variation with a geometric mean of each CPCB-2 instrument and DMPS channel from data measured on weekdays during Apr 27 to May 31, 2018. The plotted DMPS distributions are then the arithmetic mean of the average distributions measured during denoted hours of the day. Additional bins from CPCB-2 were calculated by subtracting the diurnal variations of adjacent instruments, giving concentrations in size ranges of 1.2–3 and 3–7 nm and then taking the mean of denoted hours and dividing the result with the corresponding dlog *d*_p_ value, where *d*_p_ is particle diameter.

Inside the given measurement period, the amount of valid number concentration (*N*) data varied between the instruments. The DMPS measured during the whole period with 9 min scans, whereas CPCB-2 instruments measured with 1 s resolution with following coverages: *N*_>7nm_ 80.3% (405 h), *N*_>3nm_ 61.3% (309 h), and *N*_>1.2nm_ 40.15% (202 h), subscript denoting the size range. As the subtraction of adjacent CPCB-2 instruments was done after averaging over measurement period, potential time dependency was checked by calculating the distribution also from the times when the DMPS and all CPCB-2 instruments were measuring simultaneously [27.2% (137 h), Figure S6].

The ambient distribution consisted of multiple modes, most notably a nucleation mode and an Aitken mode. The section measured with the DMPS reached a global or local peak around 55 nm during all times of the day. The peak seemed to derive from Aitken mode particles combined with an accumulation mode consisting of, e.g., soot. Concentrations increased toward smaller particle sizes in the morning and afternoon creating a distinct nucleation mode. Interestingly, the highest sub-7 nm particle concentrations were measured in the afternoon whereas the highest concentrations of particles larger than 7 nm were measured in the morning. This could highlight the role of daytime photochemical processes which typically mostly affect the nucleation mode. The influence of new particle formation during the campaign is discussed more thoroughly by Okuljar et al.^36^

### 3.2 Non-Volatile Particle Number Concentrations

**Figure 4:**
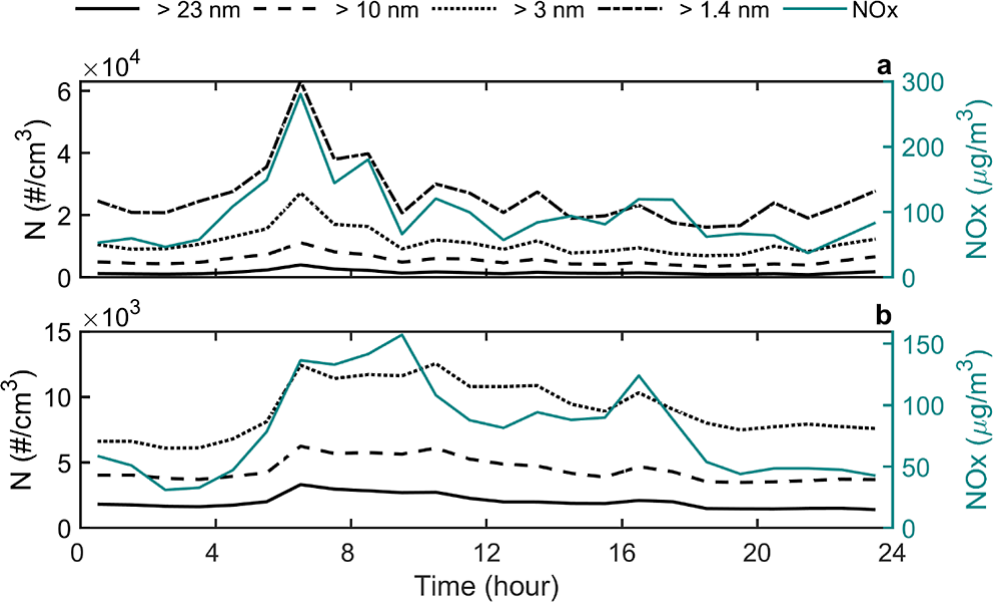
Diurnal variation of non-volatile particle number concentrations (*N*) and NO_*x*_ on weekdays during a (a) short measurement period (May 24 to 28) including sub-3 nm particles and a (b) longer measurement period (Apr 27 to May 28) for particles larger than 3 nm. Note the different y scales in the figures.

Average diurnal variation of non-volatile particle number and NO_*x*_ is presented in Figure 4 from the 3 day period including sub-3 nm size range and from a 1 month period covering only the larger size ranges of the volatility CPCB measurement.

Based on Figure 4, diurnal variations of non-volatile particle number concentration and NO_*x*_ followed relatively similar patterns suggesting that the traffic is a major source of non-volatile particle number at the measurement site. Higher concentrations measured during the 3 day period (Figure 4a) compared to the 1 month period (Figure 4b) result likely from differences in meteorological conditions as e.g., wind direction and speed as well as atmospheric mixing height can significantly alter concentrations measured in street environments.^37,38^ However, as the NO_*x*_ level was also higher and of similar shape as shown in Figure 4a, sub-3 nm non-volatile particle number concentrations also appear to originate from traffic. The difference between the periods underlines the importance of long-term measurements as an individual day may vary significantly from an average day, at least in terms of measured concentrations. During the 1 month measurement period non-volatile particle number concentrations started to elevate in the morning before 6 a.m. and were highest between 6 and 11 a.m. This corresponded to the morning rush hour and, interestingly, typical driving hours of heavy-duty vehicles which peaked also after the morning commuter traffic at the measurement site of this study (Figure S7). After this, concentrations decreased gradually apart from a moderate peak in the afternoon around 4–5 p.m. One explanation for lower concentrations in the afternoon is that the traffic flow is slightly more dominant on the opposite side of the street during afternoon rush hour. Also, the atmospheric mixing layer height and wind speed tend to be higher in the afternoon resulting in more efficient atmospheric dilution.^39^ In summertime, the mixing layer height at the Supersite typically varies from a minimum of 400 m during nighttime to a maximum of over 2000 m during the afternoon.^39^

The concentrations of non-volatile particles larger than 10 nm and larger than 3 nm were on average 2.3 and 4.6 times higher than the concentration of non-volatile particles larger than 23 nm, respectively. Thus, the number of non-volatile particles in size ranges below 23 nm clearly exceeds the number of particles larger than 23 nm. We think this observation can significantly change the current understanding of the characteristics of the smallest atmospheric particles, having implications both for the health impacts of aerosols and atmospheric effects of particles.

### 3.3 Effect of the Thermal Treatment Temperature

**Figure 5:**
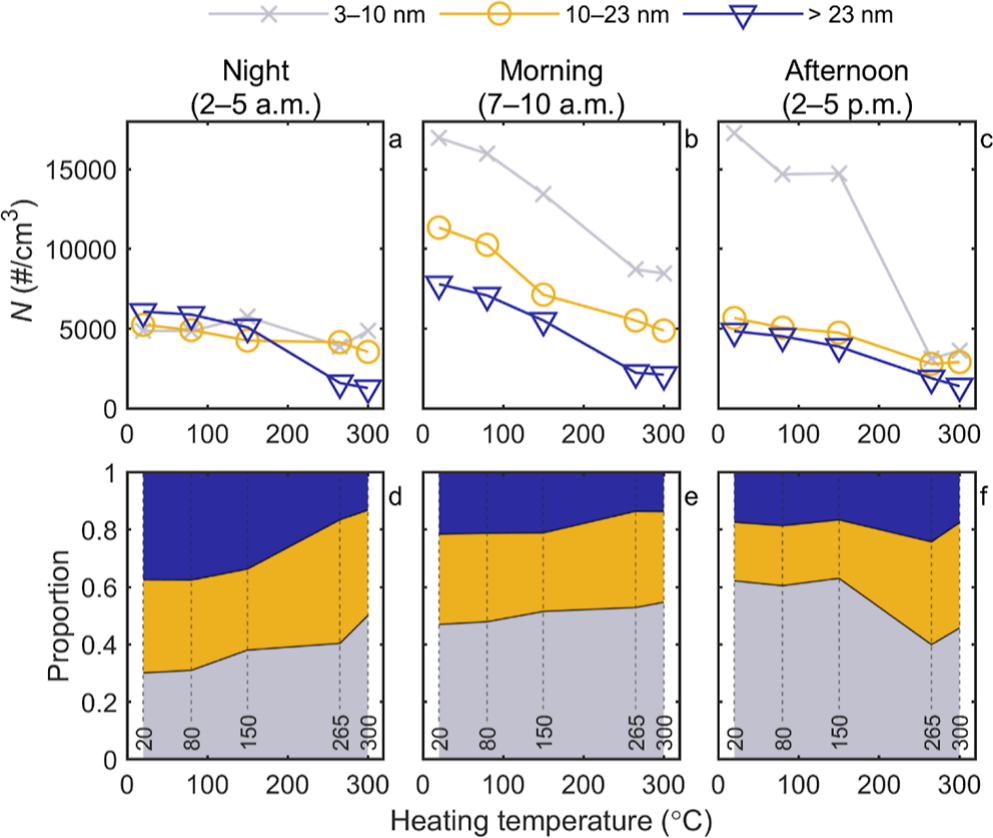
(a–c) Particle number concentration (*N*) and (d–f) proportion of particles in each particle size range in the total particle number concentration (*d*_p_ > 3 nm) as a function of thermal treatment temperature. Heating temperature corresponds to the sample temperature in the hot ejector. Data measured during night, morning, and afternoon are presented separately.

In general, the results of the removal of semivolatile components based on thermal heating were as expected: the higher the temperature was used in the hot ejector, the higher the fraction of semivolatile components was removed from particles. To study the effect of heating temperature on measured concentrations in different size ranges, the temperature of the hot ejector was varied in a cycle for 1 week as previously explained in Section 2.2. Figure 5 presents particle number concentrations of size ranges 3–10, 10–23, and >23 nm as a function of sample temperature in the hot ejector. In addition, the fraction of the number concentration in each size range to the total particle number, determined as the number concentration of particles larger than 3 nm, is shown. Data in Figure 5 were collected from the weekdays of the temperature cycle period (May 22 to 28) and different hours of the day were segregated to represent night, morning, and afternoon similarly as in Section 3.1. Similar analysis including the sub-3 nm size range is found in Figure S8.

During the night, the concentrations in general were relatively low. Without any heating, the concentrations of particles in different size ranges were almost the same regardless of the measured size range and the proportion of particles larger than 23 nm was at its largest. There was a slight concentration decrease of 10–23 nm particles as well as particles larger than 23 nm with a temperature step of 80 to 150 °C. Yet, a more notable change occurred at a step of 150 to 265 °C as the concentration of particles larger than 23 nm decreased, while the others remained relatively the same. This resulted in a decrease in proportion of particles larger than 23 nm and an increase in proportions of size ranges 3–10 nm and 10–23 nm. During the night, in general, the aerosol particles of ambient air can be more aged than in other times of the day and thus the air comprises larger particles with condensed layers of volatile and semivolatile components on their surface. Hence, average particle size decreases as the sample goes through thermal treatment and those volatile and semivolatile compounds evaporate. It should be recognized that our experiment indicates that the urban air also contains particles without any non-volatile core. This is seen as a drop of total number concentrations as a function of heating temperature.

In the morning, the concentrations in each particle size range were high (Figure 5b) and decreased relatively equally as the heating temperature increased. Especially in heating temperatures lower than 265 °C, the proportion of particles larger than 23 nm in total particle number was smaller than during the night. In our study, the busy road was just next to the measurement station, meaning that the aerosol sample was significantly contributed by relatively fresh aerosol. Interestingly, during the morning hours, the proportions of particles in different size ranges did not vary substantially as a function of heating temperature. Yet, there was an 8% decrease in the proportions of particles larger than 23 nm and, correspondingly, an increase in the proportions of 3–10 nm particles toward the highest temperatures. This suggests that a significant fraction of particles having a sub-10 nm non-volatile core are present in traffic-influenced urban environments. It should be noted that the concentrations remained high even with high heating temperatures. At 300 °C, the total particle number concentration (*d*_p_ > 3 nm) was still more than 15,000 #/cm^3^ which settles in between high particle number concentration estimates given by WHO,^13^ >10,000 #/cm^3^ (1 h mean) and >20,000 #/cm^3^ (24 h mean). Therefore, the non-volatile concentrations alone can be considerable in the mornings, in respect of WHO recommendations.

In the afternoon, the concentration of size range 3–10 nm particles started at its highest values, whereas the concentrations of the other size ranges were rather low. The latter part is most likely explained by differences in traffic patterns and meteorological conditions between morning and afternoon as already discussed in the previous section. Regarding the first part, it is of interest that the concentrations of 3–10 nm particles remained high until a steep decrease taking place at a temperature step from 150 to 265 °C. Then, the proportion of 3–10 nm particles decreased from 63 to 40%. This could indicate that a major fraction of 3–10 nm particles are formed in photochemical processes. In previous studies, the same temperature step has been related to a decrease in the concentration of particulate sulfates,^40−42^ supporting the idea that the concentration change might connect with semivolatile particles of photochemical origin. On the other hand, the particulate sulfate can be originated also from direct sulfuric acid emissions that can contribute to particle formation already in very early phases of atmospheric dilution of emitted aerosols.^26,37^ Anyway, our results showed that the urban ambient aerosol particle number was dominated by semivolatile sub-10 nm particles without non-volatile core in the afternoon.

From Figure 5, the important observation is the high number of non-volatile particles smaller than 23 nm compared to particles larger than 23 nm. If the proportions measured with temperatures 265 and 300 °C are averaged, we may roughly conclude that the current emission legislation considers only 20% of the non-volatile particle number originating from traffic emission. When the regulatory limit is shifted to 10 nm, as has been proposed, the percentage will increase to 54%. Still, it is possible that the majority of the particles emitted by traffic will remain ignored as this examination did not even include the particles in the sub-3 nm size range.

### 3.4 Emission Factors

Particle number EFs were determined for both ambient particles, i.e., particles measured without any thermal treatment, and non-volatile particles in size ranges 1.4–3, 3–10, 10–23, and >23 nm, using the approach described in Section 2.5. These EFs were determined from the data measured with the volatility CPCB when the temperature in the hot ejector diluter was less than 35 °C (ambient) and 290–330 °C (non-volatile). A distinct linear correlation between the particle number concentration and CO_2_ was observed in each case (*R*^2^ range was 0.81–0.99) even though some measurement periods were short (cycling period during May 24 to 28, Figure S9 for the non-volatile case). A longer measurement data for particles larger than 3 nm resulted to relatively similar EFs than the shorter data. All EFs are presented in Table 1 along with fraction of non-volatile particles obtained by dividing non-volatile particle EFs with corresponding ambient particle EFs.

**Table 1:** Particle Number EF of Traffic and 95% Confidence Intervals for All Ambient Particles and for Non-Volatile Particles Accompanied With the EF_non-volatile_/EF_ambient_ Ratio as a Non-volatile Fraction^a^

size range	EF_ambient_ (#/(kg fuel))	EF_non-volatile_ (#/(kg fuel))	non-volatile fraction (%)
1.4–3 nm	2.1 × 10^15^ ± 0.7 × 10^15^	6.6 × 10^14^ ± 2.5 × 10^14^	32
3–10 nm	7.4 × 10^14^ ± 2.8 × 10^14^ (6.2 × 10^14^ ± 2.6 × 10^14^)	2.3 × 10^14^ ± 3.0 × 10^14^ {2.6 × 10^14^ ± 0.3 × 10^14^}	31
10–23 nm	3.9 × 10^14^ ± 1.1 × 10^14^ (2.8 × 10^14^ ± 0.6 × 10^14^)	1.7 × 10^14^ ± 0.9 × 10^14^ {1.1 × 10^14^ ± 0.1 × 10^14^}	43
>23 nm	1.7 × 10^14^ ± 0.5 × 10^14^ (1.8 × 10^14^ ± 0.2 × 10^14^)	1.1 × 10^14^ ± 1.6 × 10^14^ {1.2 × 10^14^ ± 0.2 × 10^14^}	65

a Measurement dates are indicated with brackets: May 24–28, 2018 (May 8, 14–15, 22–28) and {Apr 27–May 28, 2018}.

Non-volatile particles are a fraction of all particles in ambient air and thus the non-volatile particle EFs were smaller than ambient EFs. Yet, there are differences in the fractions of non-volatile particles between different size ranges. It needs to be noted that here we consider only particle number EFs, and the fractions might be different for particle mass EFs. On average, one third of particles smaller than 10 nm were non-volatile, whereas this fraction increased to two thirds for particles larger than 23 nm. This supports the perception that vehicle emissions of particles larger than 23 nm contain a larger fraction of non-volatile particles than the emissions of sub-23 nm particles do. Although the fraction of non-volatile particles was smaller for sub-23 particles than for larger particles, the non-volatile particle EFs of sub-23 particles were multiple times larger than the non-volatile EFs of particles larger than 23 nm. As a result, the majority of non-volatile particles were in the sub-23 nm size range.

From Table 1, we may deduce EFs for other size ranges such as >1.4, >3, and >10 nm (Table S4). For instance, the ambient EFs for size ranges 1.4–10 and >10 nm were 2.8 × 10^15^ and 5.5 × 10^14^ #/(kg fuel) and the non-volatile EFs were 8.9 × 10^14^ and 2.8 × 10^14^ #/(kg fuel), respectively, using unrounded values. Thus, the EF of ambient sub-10 nm particles is even 5 times higher than the EF of particles larger than 10 nm. Similarly, the EF of non-volatile sub-10 nm particles is still 3 times higher than the EF of particles larger than 10 nm. This is of interest as the lower particle size limit in the European emission legislation is to be decreased to 10 nm (Euro 7). From the EF perspective, even though the change in the legislated size range doubles the amount of tracked non-volatile particles, the majority still stays undetected. Similarly, with the change of lower limit from 23 to 10 nm, the non-volatile fraction of regulated particle emission decreases from 65 to 50%. This indicates that by regulating only solid particles we are ignoring half of the traffic-emitted particles even in the considered size range.

Yet, it must be noted that the 95% confidence intervals of the determined EFs are wide, especially for shorter measurement periods. Most confidence can thus be placed on the non-volatile EFs determined from approximately 1 month time of measurements, marked with curly brackets. As these values agree well with the non-volatile EFs of the shorter period, we are confident that the magnitude of the non-volatile EF for size range 1.4–3 nm is also representative.

Ambient particle number EFs have been previously studied in the same location. Rönkkö et al.^21^ determined the ambient EF for 1–3 nm particles to be 2.6 × 10^15^ #/(kg fuel). For particle size ranges 1–3, 1–7, and >1 nm, Hietikko et al.^35^ concluded ambient EFs of 9.4 × 10^14^, 2.7 × 10^15^, and 4.2 × 10^15^ #/(kg fuel), respectively. Comparable EFs of this study are 2.1 × 10^15^, 2.8 × 10^15^, and 3.4 × 10^15^ #/(kg fuel) using size ranges 1.4–3, 1.4–10, and >1.4 nm. Thus, results are well in line with the previous studies although some variation exists likely due to temporal differences and analysis methods. It should be noted that especially the formation of volatile UFP or nucleation mode particles emitted by vehicle engines can be sensitive to ambient conditions^37,38,43,44^ and thus also the differences in ambient temperature and humidity and atmospheric mixing of emitted aerosols can affect their EFs measured in real-world conditions.

In a chase study, Järvinen et al.^45^ determined ambient EFs of Helsinki city buses following different Euro emission standards. For a Euro VI bus, EFs were 0.01 × 10^15^ and 0.19 × 10^15^ #/(kg fuel) for particles in size ranges >3 and 1.3–3 nm, respectively, whereas for a retrofit bus the EFs for the same size ranges were 4.56 × 10^15^ and 7.97 × 10^15^ #/(kg fuel). These agree with the EFs of this study but, in addition, emphasize the potentially large differences between individual vehicles in respect of the emissions of the smallest particles.

To compare the EFs of this study with the European emission standards, the EF of non-volatile particles larger than 23 nm can be converted to 7.1 × 10^12^ #/km assuming a fuel weight of 0.8 kg/L and an average fuel consumption of 8 L per 100 km. This approximation does not consider heavy-duty vehicles which would lead to a higher EF as heavy-duty vehicles have a much higher fuel consumption. The EF obtained from this study exceeds the current Euro 6 limit value of 6 × 10^11^ #/km. This is rather unsurprising since even a higher EF (1.0 × 10^14^ #/km) has been reported near a motorway.^46^ Yet, in an urban street canyon, the potential health effects caused by these high emissions may be of serious concern.

### 3.5 Limitations and Implications

When considering the results of this study, one must pay attention to the length of different measurement periods. For instance, data presented in Figure 5 were collected during a period of only 5 days, during which the heating temperature was cycled. Thus, we call for long-term ambient sub-23 nm particle number measurements which also consider particles’ volatility. As the thermal treatment is somewhat challenging especially for sub-3 nm particles, as seen also in this paper, development of both low particle loss sampling and reliable removal of all volatile compounds must continue. This study represents an urban traffic environment with relatively clean background in spring. As the sub-23 nm particles highly depend on local emissions, further research is needed in different environments such as residential areas especially when influenced by nearby air traffic. The results might also be different during another season such as a cold winter with less sunlight. In addition, detailed analyses of the effects of air flow patterns on sub-23 nm particles could provide valuable information on their formation and dispersion patterns.

This study highlights the role of UFPs in the urban air influenced by traffic and shows that the traffic emissions of particles smaller than 10 nm can be significantly higher than the emissions of particles larger than 10 nm. The particle size of 10 nm or smaller is mentioned as a lower limit in recent measurement recommendations given by WHO and e.g., the air quality directive propositions of the EU may follow those. However, decisions on the exact particle sizes to be measured have not been done yet. It is important to note that the selection of the smallest particle size to be included in particle number measurements can significantly affect the concentration results, affecting thus e.g., the comparability of air quality measurements in different cities and countries. Furthermore, our result that only a fraction of particles in these small particle sizes consist of non-volatile particles can induce additional challenges for regulatory particle number measurements; the size and the concentration of these particles can significantly depend on environmental conditions and e.g., the distance from traffic. Also, particle losses in sampling lines and a sample treatment can have a crucial role in terms of measured concentrations. E.g., in this study, the sampling line losses of sub-23 nm particles were carefully considered (see the Supporting Information) but that kind of need should be considered also in the future particle number measurement standards.

The emission regulations based on number emission of solid particles larger than 23 nm have been effective, resulting in the utilization of diesel and gasoline particle filters^47^ as well as continuously decreasing BC concentrations in urban atmospheres.^48,49^ In fact, the diesel particle filters have shown to be highly effective in removing non-volatile particles also smaller than 23 nm.^47^ Thus, it is likely that the measured non-volatile sub-23 nm particles originated mainly from vehicles without a particle filter (pre-Euro 5 or port-injection gasoline vehicles) or having a malfunctioning one. However, in this study, the non-volatile particle concentrations in traffic-influenced urban environment were dominated by sub-23 nm particles. From this perspective, the change of lower limit from 23 to 10 nm in Euro 7 is reasonable, if keeping in mind that the amount of non-volatile sub-10 nm particles measured at the curbside still exceeded the number of larger particles here. Sub-10 nm particles can also originate from brake materials,^50^ which may play a more significant role in the future as the proportion of electrical vehicles in the vehicle fleet will increase. Another challenge for emission control efforts will also remain: liquid UFPs that are formed from exhaust gases as they cool and mix in the atmosphere.^49^ Overall, there will potentially be inconsistencies in the EU air quality and emission legislation; while the particle number concentration of ambient air is expected to be monitored in the future with a lower particle size limit of approximately 10 nm, covering both non-volatile and semivolatile particles, the emission standards will only include solid particles. These differences increase the complexity of air pollution control.

## Supporting Information Available

Volatility CPCB sampling line loss correction procedure along with experimental details and results illustrating the measurement conditions and data analysis choices (PDF)

## Abbreviations

CPC: condensation particle counter
CPCB: condensation particle counter battery
DMPS: differential mobility particle sizer
DR: dilution ratio
EF: emission factor
ER: emission ratio
EU: European Union
HSY: Helsinki Region Environmental Services Authority
H/W: height-to-width ratio
PMP: particle measurement program
PM_2.5_: fine particulate matter
PSM: particle size magnifier combined with a condensation particle counter
UFPs: ultrafine particles
WHO: World Health Organization
