# Supplementary material for: Sub-23 nm Particles Dominate Non-Volatile Particle Number Emissions of Road Traffic
Source: Environ Sci Technol. 2023 Jul 14;57(29):10763–72. doi: 10.1021/acs.est.3c03221 (PMC10373488; doi:10.1021/acs.est.3c03221)
Supplement: Supplementary file 1 — es3c03221_si_001.pdf [file es3c03221_si_001.pdf]

Supporting Information to:

Sub-23 nm particles dominate non-volatile particle number emissions of road traffic

Henna Lintusaari[†,], Heino Kuuluvainen[†], Joonas Vanhanen[¤], Laura Salo[†], Harri Portin[⊥], Anssi Järvinen[†,‖], Paxton Juuti[†,∔], Riina Hietikko[†,‡], Kimmo Teinilä[§], Hilkka Timonen[§], Jarkko V. Niemi[⊥], Topi Rönkkö[†,*]*

[†]Aerosol Physics Laboratory, Physics Unit, Tampere University, Tampere, 33720, Finland

[¤]Airmodus Oy, Helsinki, 00560, Finland

[⊥]Helsinki Region Environmental Services Authority, Helsinki, 00240, Finland

[§]Atmospheric Composition Research, Finnish Meteorological Institute, Helsinki, 00560, Finland

*(H.L.) E-mail: henna.lintusaari@tuni.fi

*(T.R.) E-mail: topi.ronkko@tuni.fi

Present address: [‖]A.J.: Emission Control and Sustainable Fuels, VTT Technical Research Centre of Finland, Espoo, 02150, Finland

Present address: [∔]P.J.: Karsa Oy, Helsinki, 00560, Finland

Present address: [‡]R.H.: OptoFidelity Oy, Tampere, 33720, Finland

Document contains:

25 Pages

9 Figures

4 Tables

CONTENTS

Table S1. Measurement matrix

S1. Loss correction of the Volatility Condensational Particle Counter Battery sampling line

S1.1. Measurements

Figure S1. Measurement setup for determining line losses

Figure S2. Penetration of the sampling line

S1.2. Semi-empirical penetration fits

Table S2. Fit constants for different measurement settings

S1.3. Total penetration and loss correction

Figure S3. Illustration of the penetration coefficients

Table S3. Penetration coefficients

S1.4. Sub-3 nm particle line losses

S2. Dilution ratio of the Volatility Condensational Particle Counter Battery sampling line

Figure S4. Ambient temperature and relative humidity during the measurement campaign

S3. Data averaging

Figure S5. Illustration of particle number concentration data distribution and averaging method

Figure S6. Ambient particle number size distribution corresponding to the periods when the DMPS and the CPCB-2 instruments were measuring validly at the same time

Figure S7. Diurnal variation of vehicles passing the Supersite

Figure S8. Particle number concentration and proportion of particles in each particle size range, including also sub-3 nm size range, in the total particle number concentration as a function of thermal treatment temperature

Figure S9. Slopes for emission factor calculation of non-volatile particles during a short measurement period (24 to 28 May)

Table S4. Particle number emission factors for additional size ranges

References

Table S1. Measurement matrix. Note that the label "instrument measuring" does not guarantee a full 24 h measurement day.

Date		Volatility Condensation Particle Counter Battery (Volatility CPCB)				Additional measurements					
						CPCB-2					
		Hot ejector mode	Sample temperature in hot ejector (°C)	PSM Saturator flow (lpm)	Notes	A20 CPC	CPC 3776	A11 nCNC	DMPS	NOx	CO2
Fri	27.4.2018	fixed	300	1.3		x	x	x	x	x	x
Sat	28.4.2018	fixed	300	1.3		x	x	x	x	x	x
Sun	29.4.2018	fixed	300	1.3		x	x	x	x	x	x
Mon*	30.4.2018	fixed	300	1.3		x	x	x	x	x	x
Tue*	1.5.2018	fixed	300	1.3		x		x	x	x	x
Wed	2.5.2018	fixed	300	1.3		x		x	x	x	x
Thu	3.5.2018	fixed	300	1.3		x	x		x	x	x
Fri	4.5.2018	fixed	300	1.3		x	x	x	x	x	x
Sat	5.5.2018	fixed	300	1.3		x	x	x	x	x	x
Sun	6.5.2018	fixed	300	1.3		x	x	x	x	x	x
Mon	7.5.2018	fixed	300	1.3		x		x	x	x	x
Tue	8.5.2018	fixed	300	1.3		x	x	x	x	x	x
Wed	9.5.2018	fixed	300	1.3		x	x	x	x	x	x
Thu*	10.5.2018	fixed	300	1.3		x	x		x	x	x
Fri*	11.5.2018	fixed	300	1.3		x	x		x	x	x
Sat	12.5.2018	fixed	300	1.3		x	x		x	x	x
Sun	13.5.2018	fixed	300	1.3		x	x		x	x	x
Mon	14.5.2018	fixed	20	1.3		x	x		x	x	x
Tue	15.5.2018	fixed	20	1.3		x	x		x	x	x
Wed	16.5.2018	fixed	300	1.0	DR	x	x		x	x	x
Thu	17.5.2018	fixed	300	1.0		x	x	x	x	x	x
Fri	18.5.2018	fixed	300	1.0		x	x	x	x	x	x
Sat	19.5.2018	fixed	300	1.0		x		x	x	x	x
Sun	20.5.2018	fixed	300	1.0		x		x	x	x	x
Mon	21.5.2018	fixed	300	1.0		x	x	x	x	x	x
Tue	22.5.2018	cycle	20–300	1.0	TS	x			x	x	x
Wed	23.5.2018	cycle	20–300	1.0					x	x	x
Thu	24.5.2018	cycle	20–300	1.0		x	x	x	x	x	x
Fri	25.5.2018	cycle	20–300	1.0	TS	x	x	x	x	x	x
Sat	26.5.2018	cycle	20–300	1.0		x	x	x	x	x	x
Sun	27.5.2018	cycle	20–300	1.0		x	x		x	x	x
Mon	28.5.2018	cycle	20–300	1.0	DR	x	x	x	x	x	x
Tue	29.5.2018	-	-	-		x	x	x	x	x	x
Wed	30.5.2018	-	-	-		x	x	x	x	x	x
Thu	31.5.2018	-	-	-		x	x		x	x	x

* A holiday or a day between a holiday and a weekend
x = Instrument measuring
DR = Dilution ratio measurement
TS = Temperature step measurement

S1. Loss correction of the Volatility Condensational Particle Counter Battery sampling line

S1.1. Measurements

Losses of the Volatility CPCB sampling line were measured in a laboratory by producing 5-50 nm silver particles with a tube furnace and measuring the number concentration upstream and downstream of the sampling line. A tube furnace has previously been used to generate silver particles, e.g., by Harra et al.[1]. Line losses of the flow splitter, and the tubing after, were measured separately from the rest of the sample line for characterization reasons.

The measurement setup for line losses before the flow splitter is presented in Figure S1. The setup comprised of a generation of the sample aerosol, a measurement of the sample distribution, a measurement of the dilution ratio, and a measurement of the actual losses. As the temperature of the tube furnace affects the size distribution of generated particles, the distribution was first studied with a combination of a Nano Differential Mobility Analyzer (Nano DMA, TSI 3085A) and a CPC (TSI 3756) used in scanning mode. DMA was connected to an Electrostatic Classifier (TSI 3082) which included a neutralizer and provided the needed flows as well as controlled the DMA current. A sheath flow of 15 lpm or 20 lpm was used in the measurements.

A nitrogen flow was introduced through the tube furnace and the sample was diluted after the furnace with additional nitrogen to keep particle number concentrations and a sample temperature suitable for the CPC. The tube furnace was used with temperature range from 1000 to 1200 ºC. The temperature was adjusted to provide as unimodal, lognormal distribution as possible, when the desired particle size was selected with the Nano DMA from the right side of the distribution. This way the amount of multiply charged particles was minimized and generated particles were treated as singly charged. After the optimal generation temperature for a certain

particle size was found, the measurement could continue with the loss measurements of that particle size since the generation was measured to be stable.

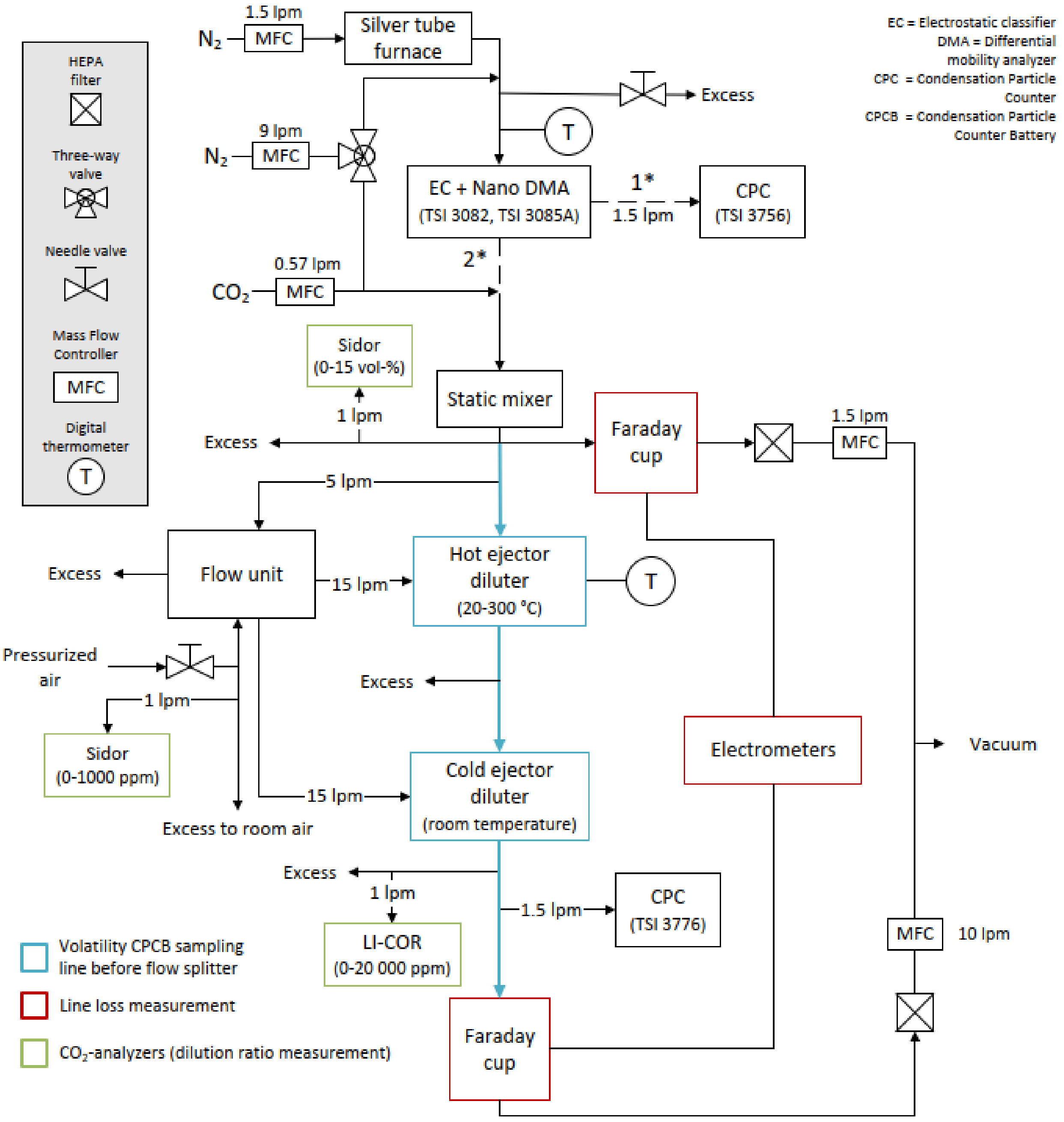

Distribution measurement: line 1* in use, line 2* off, dilution before DMA, excess needle valve open.
Line loss measurement: line 2* in use, line 1* off, dilution after DMA, excess needle valve closed.

Figure S1. Measurement setup for measuring line losses of the Volatility Condensational Particle Counter Battery sampling line before the flow splitter.

When measuring line losses, the DMA current was fixed to correspond with the particle size in question. Thus, the sample comprised of mainly singly charged particles of certain size after the DMA. The sample was diluted only after the DMA with both nitrogen and CO_2, latter to measure the dilution ratio of the sample line simultaneously. The sample flow was split after a static mixer to a Faraday Cup Aerosol Electrometer (FCAE), the sampling line of the Volatility CPCB, and a CO_2 analyzer (SICK SIDOR, 0-15 %). Similar split was done after the Volatility CPCB sampling line to another FCAE, a CPC (TSI 3776), and a CO_2 analyzer (LI-COR LI-840A, 0-20 000 ppm). A third CO_2 analyzer (SICK SIDOR, 0-1000 ppm) was measuring the background CO_2 concentration of the dilution air used in ejectors.

The number concentration before and after the sampling line was determined with FCAEs as explained in Yli-Ojanperä[2, P. 4-5] using equation

$$\boldsymbol{N} = \frac{\boldsymbol{\Delta I}}{\boldsymbol{Qne\eta_{\mathrm{FCAE}}\gamma}}, \qquad (2.1)$$

where $\boldsymbol{\Delta I}$ is the offset corrected current measured by FCAE, $\boldsymbol{Q}$ is the volumetric flow rate of the cup, $\boldsymbol{n}$ is the average charge of the particles, $\boldsymbol{e}$ is the elementary charge, $\boldsymbol{\eta_{\mathrm{FCAE}}}$ is the detection efficiency of the FCAE and $\boldsymbol{\gamma}$ is the calibration factor of the electrometer. The offset corrected current was calculated as

$$\boldsymbol{\Delta I} = \boldsymbol{I_{\mathrm{particle}}} - \frac{\boldsymbol{I_{\mathrm{offset-}}} + \boldsymbol{I_{\mathrm{offset+}}}}{\mathbf{2}}, \qquad (2.2)$$

where $\boldsymbol{I}_{\textbf{particle}}$ is the average current induced by the particles, $\boldsymbol{I}_{\textbf{offset}-}$ is the measured background signal current before particles are introduced, and $\boldsymbol{I}_{\textbf{offset}+}$ is the measured background signal current after the particles. During the measurements, the DMA current was periodically turned off for a minute and again on for a minute to avoid the effects of potentially drifting background signal. The CPC downstream of the sampling line was used to verify that no particles were present when the DMA current was off, meaning that the measured FCAE background signal was an absolute zero level.

Dilution ratio (DR) of the sampling line was determined from the ratio of the CO_2 analyzers upstream and downstream of the sampling line, taking into account the background CO_2 level. Penetration could thus be measured using equation

$$\boldsymbol{P} = \frac{\boldsymbol{N}_{\textbf{out}}}{\boldsymbol{N}_{\textbf{in}}} = \frac{\frac{\Delta \boldsymbol{I}_{\textbf{out}}}{\boldsymbol{Q}_{\textbf{out}} \boldsymbol{ne}\boldsymbol{\eta}_{\textbf{FCAE}}\boldsymbol{\gamma}}}{\frac{\Delta \boldsymbol{I}_{\textbf{in}}}{\boldsymbol{Q}_{\textbf{out}} \boldsymbol{ne}\boldsymbol{\eta}_{\textbf{FCAE}}\boldsymbol{\gamma}}} \textbf{DR} = \frac{\Delta \boldsymbol{I}_{\textbf{out}}}{\Delta \boldsymbol{I}_{\textbf{in}}} \frac{\boldsymbol{Q}_{\textbf{in}}}{\boldsymbol{Q}_{\textbf{out}}} \textbf{DR}, \quad (2.3)$$

where subscripts “in” and “out” correspond to upstream FCAE and downstream FCAE, respectively. FCAE detection efficiencies and electrometer calibration factors were reduced since both cups and electrometers were identical and thus their characteristics were approximated to be the same. A higher flow rate (10 lpm) was used in the downstream FCAE to increase the signal current since the sample was diluted in the Volatility CPCB sampling line.

The final penetrations were averaged from the multiple one-minute measurements. These are presented in Figure S2 as a function of particle size. The standard errors were less than 1 % for all particle sizes except 5 nm which had standard error less than 3 %. However, this does not consider the error caused by the FCAE flow rate variations and measurements, which was estimated to be at maximum 6 %.

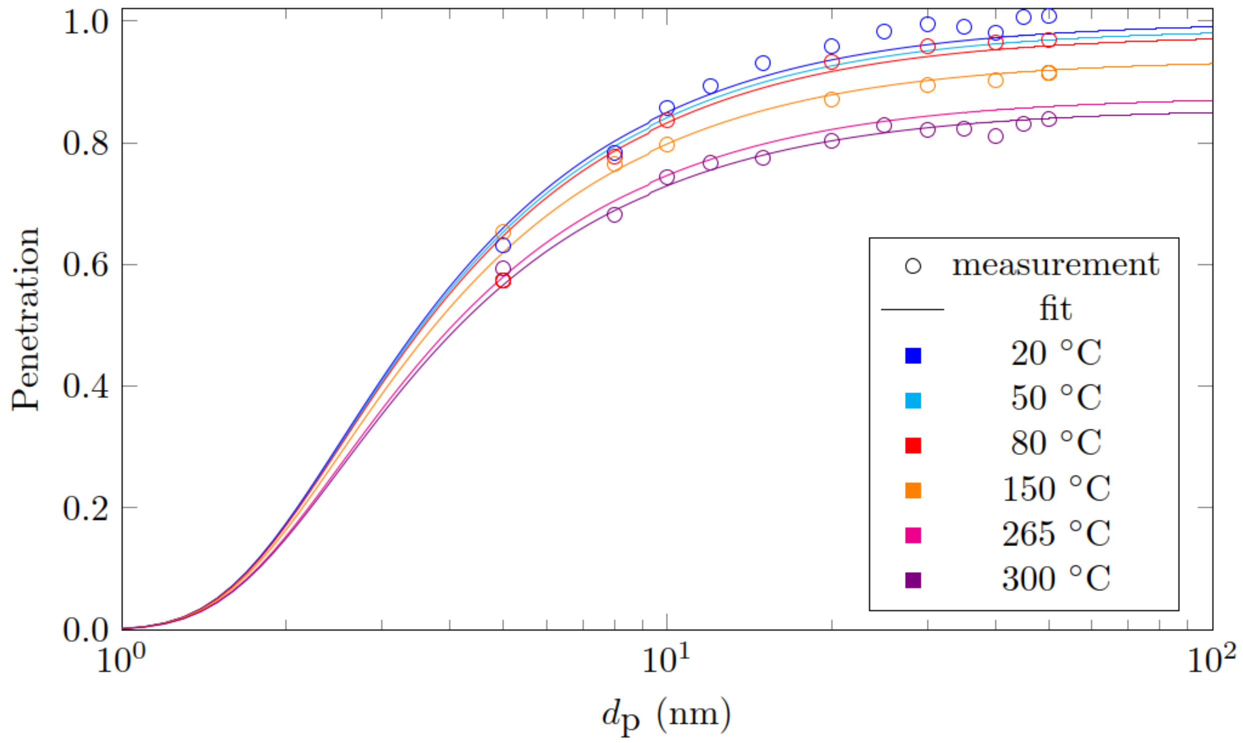

Figure S2. Penetration of the Volatility Condensational Particle Counter Battery sampling line before the flow splitter as a function of particle diameter d_p. Temperatures indicate the heating temperature of the 1st ejector diluter in line.

S1.2. Semi-empirical penetration fits

Fits for the measurement points were derived using the least squares method. Diffusion and thermophoresis were assumed to be the causes for the significant losses. Thus, an expression was derived using the theoretical equations of these two phenomena. In size range of 5 to 50 nm, thermophoretic losses are independent of the particle size and so the thermophoretic penetration $\boldsymbol{\eta}_{\mathbf{therm}}$ was considered as a constant term. Yet, this term varied between different heating temperatures of the 1st ejector. Diffusional penetration $\boldsymbol{\eta}_{\mathbf{diff}}$ was calculated using formulation of Gormley & Kennedy[3] for the transport efficiency in laminar tube flow for particles undergoing diffusive deposition as

$$\eta_{\mathrm{diff}} = 1 - 2.56\xi^{\left(\frac{2}{3}\right)} + 1.2\xi + 0.177\xi^{\left(\frac{4}{3}\right)} \quad (2.4a)$$

for $\xi < 0.02$ and

$$\eta_{\mathrm{diff}} = 0.819\exp(-3.657\xi) + 0.097\exp(-22.3\,\xi) + 0.032\exp(-57\,\xi)$$

for $\xi > 0.02$. (2.4b)

Here, the ξ is calculated from equation

$$\xi = \frac{\pi D L}{Q}, \quad (2.5)$$

where D is particle diffusion coefficient, L is tube length, and Q is volumetric flow rate through the tube[4 P. 90]. Diffusion coefficient can be expressed as

$$\mathbf{D} = \frac{k_{\mathrm{B}} T C_{\mathrm{c}}}{3\pi \mu_{\mathrm{g}} d_{\mathrm{p}}}, \quad (2.6)$$

where k_{B} is Boltzmann constant, T is temperature, C_{c} is Cunningham slip correction factor, μ_{g} is dynamic viscosity of the gas and d_{p} is particle diameter (Hinds 1999, p.153). The equation for the Cunningham slip correction factor is

$$C_{\mathrm{c}} = 1 + \frac{\lambda}{d_{\mathrm{p}}}\left(2.34 + 1.05\exp\left(-0.39\frac{d_{\mathrm{p}}}{\lambda}\right)\right), \quad (2.7)$$

where λ is the mean free path of the gas molecule[5, P. 49]. The mean free path for air molecule is in standard conditions (T = 293.15 K, p = 1 atm) is approximately 66 nm[5, P. 448]. This value was used in calculations.

As diffusional penetration was dependent on particle size, the term $\boldsymbol{\xi}$ was split to size-dependent and size-independent parts. The size-independent part was handled as one constant term $\boldsymbol{a}$ that was then multiplied with the size-dependent terms. Thus, the penetration formula for the fits was

$$\boldsymbol{P} = \boldsymbol{\eta}_{\mathbf{therm}}\boldsymbol{\eta}_{\mathbf{diff}}(\boldsymbol{\xi}) = \boldsymbol{\eta}_{\mathbf{therm}}\boldsymbol{\eta}_{\mathbf{diff}}\left(\boldsymbol{a}\frac{C_{\mathrm{c}}(\boldsymbol{d}_{\mathbf{p}})}{\boldsymbol{d}_{\mathbf{p}}}\right). \tag{2.8}$$

Fits were derived by altering variables $\boldsymbol{\eta}_{\mathbf{therm}}$ and $\boldsymbol{a}$ with the least squares method. In the optimization, the term $\boldsymbol{\eta}_{\mathbf{therm}}$ was set to stay in the range of 0-1 as is physically reasonable. The fits were solved first for each measurement temperature separately. Then the optimization was repeated for thermophoretic penetration using an average of obtained terms $\boldsymbol{a}$ as a constant. Obtained values for $\boldsymbol{\eta}_{\mathbf{therm}}$ and $\boldsymbol{a}$ are presented in Table S2.

Table S2. Fit constants obtained for different measurement settings. Term $\boldsymbol{a}_{\mathbf{0}}$ represents the size-independent part of diffusional penetration determined at first round of optimization. Final fits used the values listed under $\boldsymbol{a}$ in diffusional and $\boldsymbol{\eta}_{\mathbf{therm}}$ in thermophoretic penetration calculation.

Measurement setting	$\boldsymbol{\eta}_{\mathbf{therm}}$	$\boldsymbol{a}_{\mathbf{0}}$	$\boldsymbol{a}$
heating off	$\mathbf{1.000}$	$\mathbf{7.57 \cdot 10^{-12}}$	$\mathbf{7.51 \cdot 10^{-12}}$
80 ºC	$\mathbf{0.980}$	$\mathbf{1.03 \cdot 10^{-11}}$	$\mathbf{7.51 \cdot 10^{-12}}$
150 ºC	$\mathbf{0.939}$	$\mathbf{5.97 \cdot 10^{-12}}$	$\mathbf{7.51 \cdot 10^{-12}}$
300 ºC	$\mathbf{0.858}$	$\mathbf{6.22 \cdot 10^{-12}}$	$\mathbf{7.51 \cdot 10^{-12}}$
50 ºC	$\mathbf{0.990}$	−	$\mathbf{7.51 \cdot 10^{-12}}$
265 ºC	$\mathbf{0.878}$	−	$\mathbf{7.51 \cdot 10^{-12}}$
Flow splitter, 1.0 lpm through a branch	−	−	$\mathbf{2.56 \cdot 10^{-12}}$
Flow splitter, 2.5 lpm through a branch	−	−	$\mathbf{1.46 \cdot 10^{-12}}$

For temperatures 50 ºC and 265 ºC, as these temperatures were not included in the measurements, the thermophoretic penetration was determined assuming that the thermophoretic penetration was dependent on temperature as

$$\boldsymbol{\eta}_{\mathbf{therm}} \propto \boldsymbol{T}_{\mathbf{w}}(\boldsymbol{T}_{\mathbf{e}} - \boldsymbol{T}_{\boldsymbol{w}}),$$

where $\boldsymbol{T}_{\mathbf{w}}$ is the wall temperature (293,15 K) and $\boldsymbol{T}_{\mathbf{e}}$ is the temperature at tube entrance (simplification of an equation presented by Walker et al.[6,7]). Thus, we used the thermophoretic penetrations obtained for temperatures 20 ºC, 80 ºC, 150 ºC, and 300 ºC and their corresponding terms of $\boldsymbol{T}_{\mathbf{w}}(\boldsymbol{T}_{\mathbf{e}} - \boldsymbol{T}_{\boldsymbol{w}})$ to create a linear fit ($R^2 > 0.99$) from which the thermophoretic penetrations for temperatures 50 ºC and 265 ºC were retrieved.

S1.3. Total penetration and loss correction

Line losses in the flow splitter and tubing after were similarly determined. As the section did not include any dilution, the DR = 1 was applied in Eq. 2.3. Penetration through the flow splitter was measured for two branch flows, 1 lpm and 2.5 lpm, to match different inlet flows of the CPCB instruments. The standard error of the measured penetration was less than 1 % for all particle sizes and the error caused by FCAE flow rate variations and measurements was less than 2 % at maximum. Fits for the measurements were applied without thermophoretic term.

Total penetration through the Volatility CPCB sampling line was thus calculated as a product of the penetrations though different sections

$$\boldsymbol{P}_{\mathbf{tot}} = \boldsymbol{\eta}^{\mathbf{1}}_{\mathbf{therm}}\boldsymbol{\eta}^{\mathbf{1}}_{\mathbf{diff}}\boldsymbol{\eta}^{\mathbf{2}}_{\mathbf{diff}}, \qquad (2.9)$$

where the superscript 1 refers to the section before the flow splitter and 2 to the section after the flow splitter. As the losses depended on particle size, the correction was applied separately to different size ranges of the Volatility CPCB. The penetration for size ranges 1.4-3 nm, 3-10 nm, and 10-23 nm corresponded to the geometric mean of the size range ends. For particles larger than 23 nm, the penetration corresponded to the particle size of 55 nm, which was derived from the local maximum of the particle number distribution measured with DMPS (Fig. 2). Illustration of the approach is shown in Figure S3.

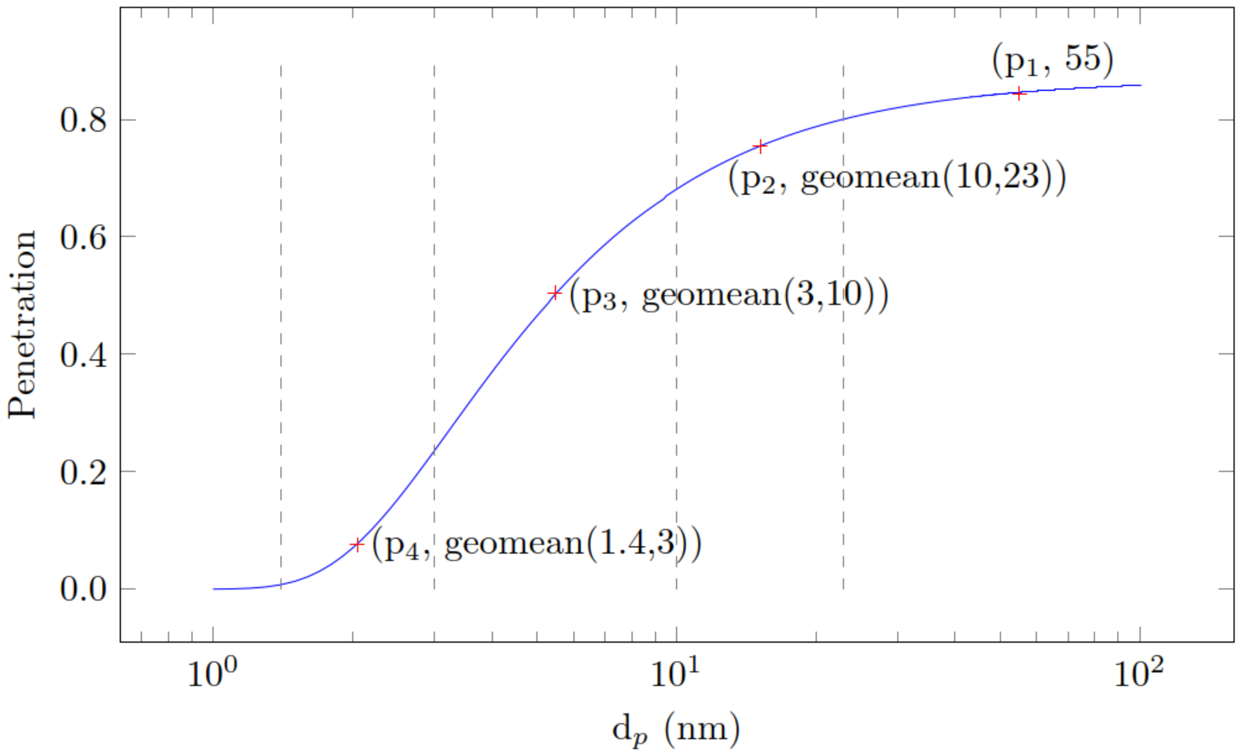

Figure S3. Illustration of how the penetration coefficients p1, p2, p3, and p4 for different particle size ranges are derived for the Volatility CPCB line loss correction. Notation geomean(dp1, dp2) denotes the geometric mean of particle diameters dp1 and dp2.

Penetration coefficients determined for each particle size range at used temperatures are listed in Table S3. Compared to the typical line losses when the line includes a Volatile Particle Remover or a Catalytic Stripper[8–10], the penetration coefficients with temperature 300 ºC are great.

Table S3. Penetration coefficients for different size ranges and heating temperatures of the Volatility CPCB sampling line. Notations p1, p2, p3, and p4 correspond to the penetration of particle size ranges > 23 nm, 10–23 nm, 3–10 nm, and 1.4–3 nm, respectively.

Heating temperature of the 1. ejector	Penetration coefficient			
	p1	p2	p3	p4
heating off	0.97	0.87	0.60	0.12
50 ºC	0.96	0.86	0.59	0.12
80 ºC	0.95	0.85	0.59	0.12
150 ºC	0.91	0.82	0.56	0.11
265 ºC	0.85	0.76	0.53	0.10
300 ºC	0.83	0.75	0.51	0.10

The number concentrations measured by the Volatility CPCB were thus corrected with the inverse of these coefficients as follows:

$$\boldsymbol{N}_{>23\text{ nm}} = \frac{1}{p_1}\boldsymbol{N}^{o}_{>23\text{ nm}} \quad (2.10)$$

$$\boldsymbol{N}_{>10\text{ nm}} = \frac{1}{p_1}\boldsymbol{N}^{o}_{>23\text{ nm}} + \frac{1}{p_2}\boldsymbol{N}^{o}_{10-23\text{ nm}} \quad (2.11)$$

$$\boldsymbol{N}_{>3\text{ nm}} = \frac{1}{p_1}\boldsymbol{N}^{o}_{>23\text{ nm}} + \frac{1}{p_2}\boldsymbol{N}^{o}_{10-23\text{ nm}} + \frac{1}{p_3}\boldsymbol{N}^{o}_{3-10\text{nm}} \quad (2.12)$$

$$\boldsymbol{N}_{>1.4\text{ nm}} = \frac{1}{p_1}\boldsymbol{N}^{o}_{>23\text{ nm}} + \frac{1}{p_2}\boldsymbol{N}^{o}_{10-23\text{ nm}} + \frac{1}{p_3}\boldsymbol{N}^{o}_{3-10\text{nm}} + \frac{1}{p_4}\boldsymbol{N}^{o}_{1.4-3\text{nm}}, \quad (2.13)$$

where $\boldsymbol{N}$ is corrected particle number concentration, $\boldsymbol{N}^{o}$ is uncorrected particle number concentration, and subscript denotes the size range.

S1.4. Sub-3 nm particle line losses

As seen from Table S3, the average penetration of 1.4–3 nm particles at 300 ºC is 0.1. Given that the PSM can detect half of the 1.4 nm particles by the definition of a cut-off size, it is evident that this size range possesses considerable uncertainties. Even though it is likely that most of the penetrated particles in this size range are rather 3 nm than 1.4 nm in size, this study follows the common practice and states the cut-off size of the instrument as the lower limit of the size range.

S2. Dilution ratio of the Volatility Condensational Particle Counter Battery sampling line

Dilution was essential to cool down the sample before the instruments and to reduce partial vapor pressures to avoid re-nucleation. The dilution ratio (DR) of the ejector diluters was measured twice during the measurement campaign to detect potential changes in DR caused by contamination in the ejectors. DR was determined by measuring a known concentration of carbon dioxide in nitrogen gas. The DR of the sampling line depended slightly on the temperature of the hot ejector increasing from 33 to 38 with a decrease of temperature from 300 to 20 ºC.

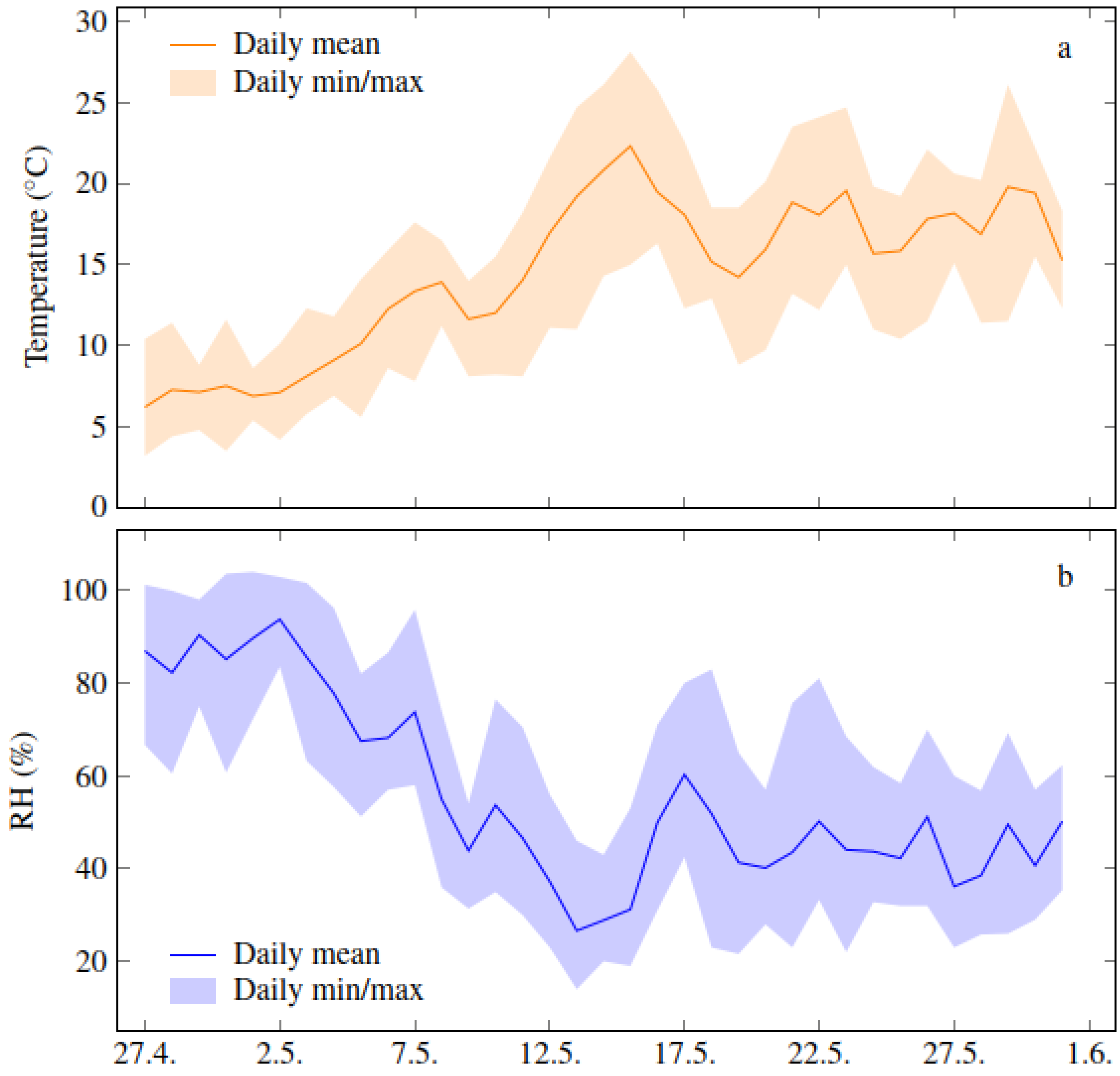

Figure S4. Ambient a) temperature and b) relative humidity (RH) during the measurement campaign. Temperature is measured at the Supersite and relative humidity is measured at a nearby weather station Pasila at rooftop level. Data from Helsinki Region Environmental Services.

S3. Data averaging

Particle number concentrations can vary greatly within a few seconds at a kerbside. This is due to constantly changing nearby emission sources as, e.g., different kinds of vehicles pass by. When studying the distribution of the data, we noted that the measured particle concentrations were not normally distributed but rather followed log-normal fits. An arithmetic mean tended to overestimate the averages (see Figure S5). This was especially distinct when considering ambient sub-23 nm particle concentrations. Therefore, a geometric mean was used when averaging the particle number concentrations. As zero is not mathematically defined with geometric mean and negative concentrations are not physically possible, only concentrations > 0 $\#/cm^3$ were included in the derived averages. The Volatility CPCB data contained some negative values due to the calculation method using subtraction of different size ranges. However, the number of negative values was negligible as the proportion of concentrations ≤ 0 $\#/cm^3$ was at most ~1 %.

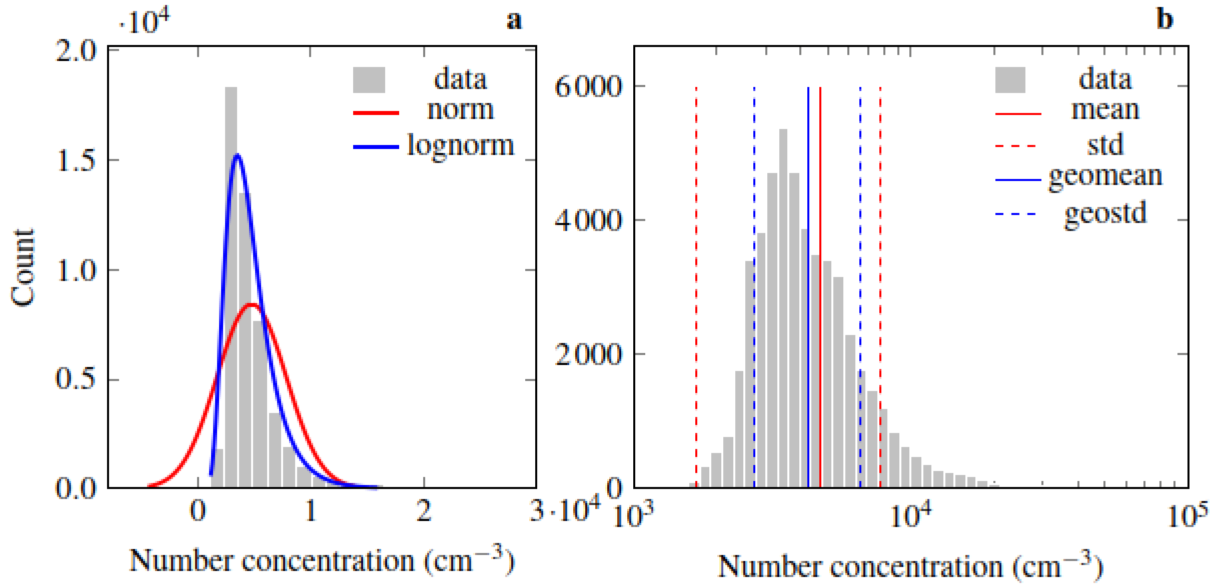

Figure S5. Distribution of particle ($d_p \geq 10$ nm) number concentrations measured between 2–3 p.m. during the period from 27 April to 13 May 2018 on (a) a linear and (b) a logarithmic x-axis. (a) Fits for a normal distribution (norm) and a log-normal distribution (lognorm) are applied and (b) both arithmetic mean (mean) and standard deviation (std), and geometric mean (geomean) and standard deviation (geostd) are calculated.

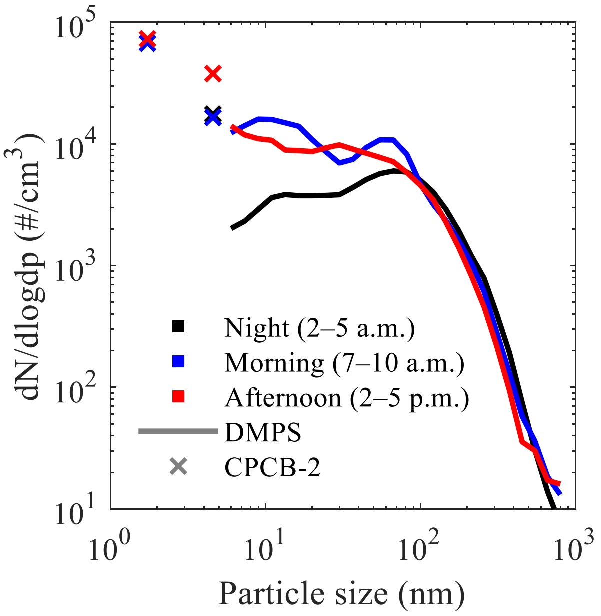

Figure S6. Ambient particle number size distribution in street canyon on weekdays. Three different times of the day, night (black), morning (blue), and afternoon (red) are distinguished from the dataset. Dataset covers 27.2 % of the weekdays during the measurement period (27 Apr to 31 May 2018) corresponding to the periods when the DMPS and the CPCB-2 instruments were measuring validly at the same time. Geometric mean used. Note that the smallest size bin is missing during night, since the concentrations measured with size ranges > 3 nm and > 1.2 nm were close to each other causing a slightly negative value for the calculated concentration in size bin 1.2–3 nm. This was the only significant difference compared to Figure 3 and indicates that the biggest uncertainty lies in this size range during the night.

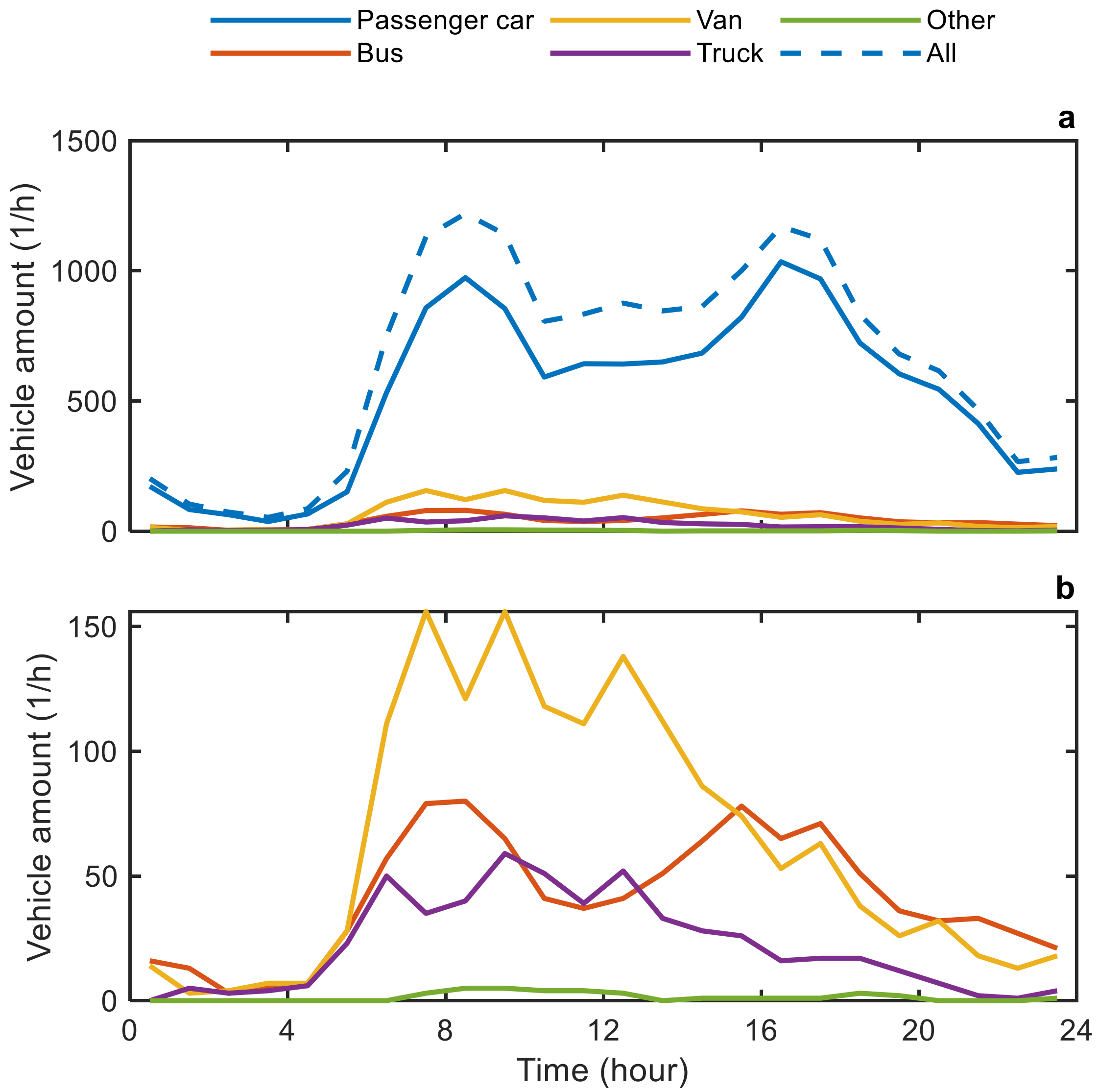

Figure S7. Diurnal variation of a) all vehicles and b) vehicles excluding passenger cars passing the Supersite on the same side of the street. Data collected by visual classification from videos recorded on 9 May 2017 from 10.30 to 11.30 a.m. (1h) and from 10 May 2017 11.30 a.m. to 11 May 2017 10.30 a.m. (23 h).

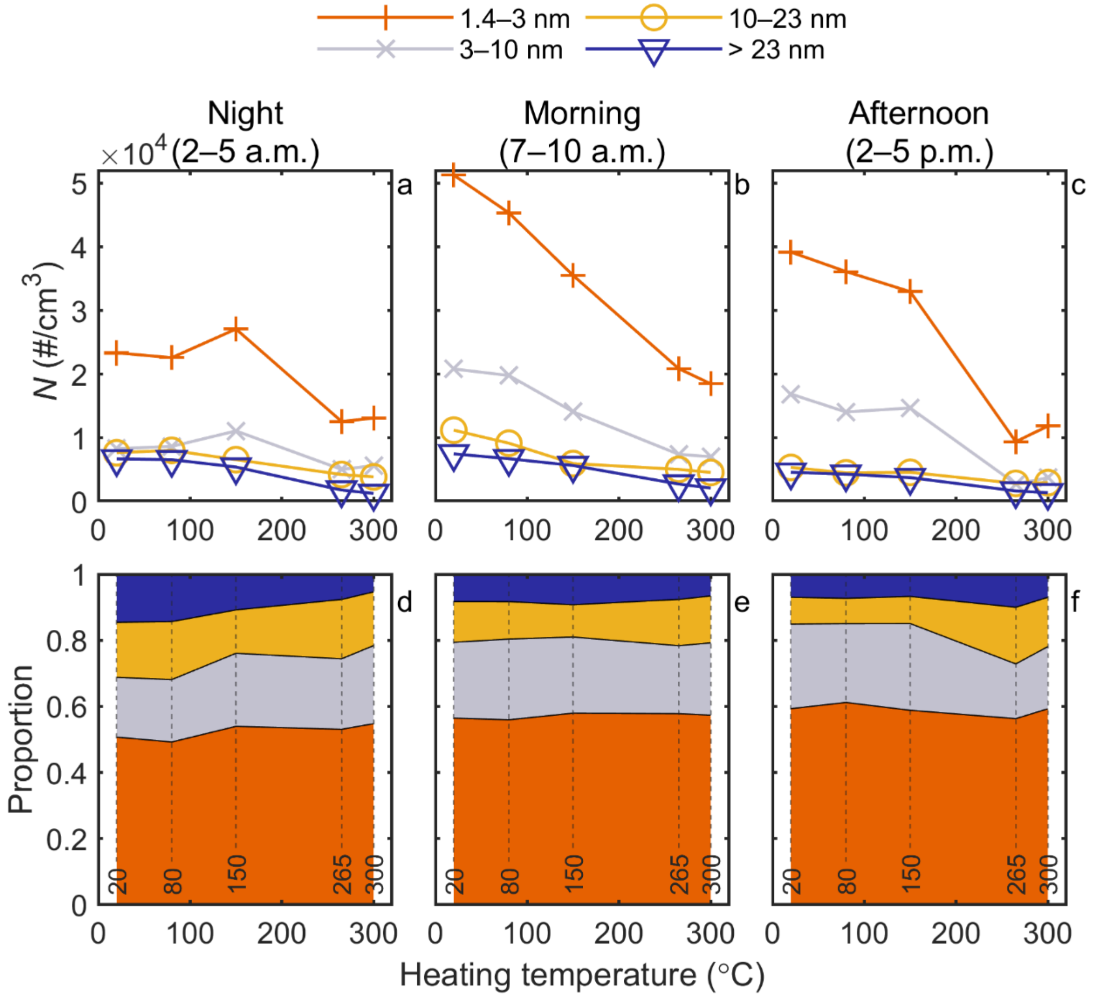

Figure S8. (a-c) Particle number concentration (N) and (d-f) proportion of particles in each particle size range in the total particle number concentration (d_p > 1.4 nm) as a function of thermal treatment temperature. Heating temperature corresponds to the sample temperature in the hot ejector. Data measured during night, morning, and afternoon are presented separately. Data was collected during a three-day period of temperature cycle type measurement from 24 to 28 May 2018. Note the high uncertainties in the sub-3 nm size range due to the limited amount of data, the potential for a small artefact, and significant line losses.

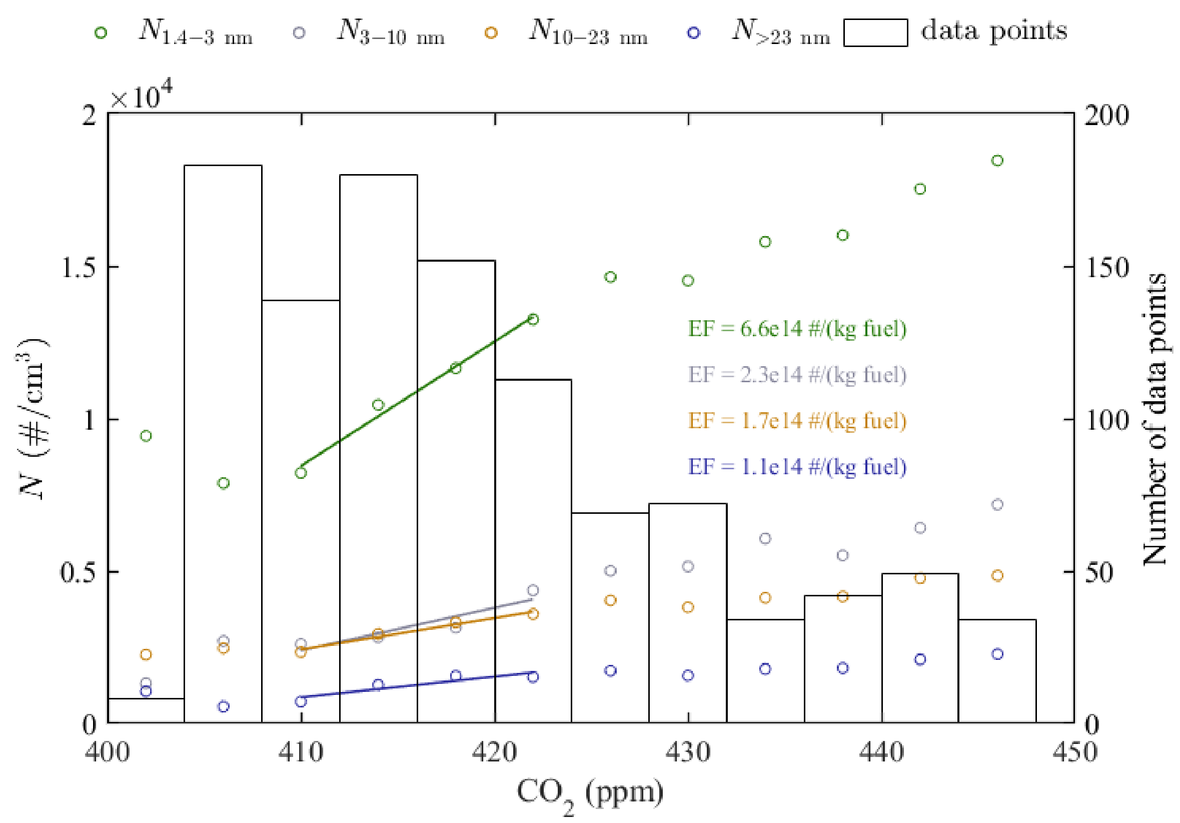

Figure S9. Particle number concentration (N) of non-volatile particles in different size ranges as a function of simultaneously measured CO_2 concentration. Circular markers represent averages of number concentrations in 4 ppm CO_2 intervals. Right y-axis represents the number of data points in each CO_2 interval in the histogram. Linear fits are applied to markers with CO_2 values above background level and more than 100 data points. Emission factors (EF) are calculated using the slope of the fits as the emission ratio. Data has been collected during a short measurement period (24 to 28 May).

Table S4. Particle number emission factors (EF) of traffic for all ambient particles and for non-volatile particles accompanied with $EF_{non\text{-}volatile}$ / $EF_{ambient}$ ratio as a non-volatile fraction. Deduced by summing the EFs presented in Table 1, using unrounded values.

**Size range**	**$EF_{ambient}$ (# / (kg fuel))**	**$EF_{non\text{-}volatile}$ (# / (kg fuel))**	**Non-volatile** fraction **(%)**
**> 1.4 nm**	3.4×10^{15}	1.2×10^{15}	35
**> 3 nm**	1.3×10^{15} (1.1×10^{15})	5.0×10^{14} {5.0×10^{14}}	39
**> 10 nm**	5.5×10^{14} (4.6×10^{14})	2.8×10^{14} {2.3×10^{14}}	50
**> 23 nm**	1.7×10^{14} (1.8×10^{14})	1.1×10^{14} {1.2×10^{14}}	65

Measurement dates are indicated with brackets:
24–28 May 2018, (8, 14–15, 22–28 May) and {27 Apr – 28 May 2018}

References

1. Harra, J., Mäkitalo, J., Siikanen, R., Virkki, M., Genty, G., Kobayashi, T., Kauranen, M. & Mäkelä, J. M. Size-controlled aerosol synthesis of silver nanoparticles for plasmonic materials. *J. Nanoparticle Res.* **14,** 870 (2012).
2. Yli-Ojanperä, J. *Calibration of Aerosol Instruments in a Wide Particle Size Range*. (Tampere University of Technology, 2012). at <https://trepo.tuni.fi/handle/10024/114942>
4. Kulkarni, P., Baron, P. A. & Willeke, K. *Aerosol measurement: principles, techniques, and applications*. (Wiley, 2011).
5. Hinds, W. C. *Aerosol Technology: Properties, Behavior, and Measurement of Airborne Particles, 2nd Edition*. (Wiley, 1999). at <https://www.wiley.com/en-ie/Aerosol+Technology%3A+Properties%2C+Behavior%2C+and+Measurement+of+Airborne+Particles%2C+2nd+Edition-p-9781118591970>
6. Walker, K. L., Homsy, G. M. & Geyling, F. T. Thermophoretic deposition of small particles in laminar tube flow. in *J. Colloid Interface Sci.* **69,** 138–147 (1979).
7. Lin, J.-S. & Tsai, C.-J. Thermophoretic deposition efficiency in a cylindrical tube taking into account developing flow at the entrance region. *J. Aerosol Sci.* **34,** 569–583 (2003).
8. Andersson, J., Giechaskiel, B., Munoz, B. R., Sandbach, E. & Dilara, P. Particle Measurement Programme (PMP) Light-duty Inter-laboratory Correlation Exercise (ILCE_LD) Final Report. *JRC Publ. Repos.* (2007). at <https://publications.jrc.ec.europa.eu/repository/handle/JRC37386>
9. Swanson, J. & Kittelson, D. Evaluation of thermal denuder and catalytic stripper methods for solid particle measurements. *J. Aerosol Sci.* **41,** 1113–1122 (2010).
